# Assisting Employers of Small and Medium-Sized Enterprises (SMEs) to Support Employees on Long-Term Sick-Leave: Development of a Web-Based SME Tool Using Intervention Mapping

**DOI:** 10.1007/s10926-025-10281-8

**Published:** 2025-03-15

**Authors:** Donna C. E. Beerda, Frederieke G. Schaafsma, Sietske J. Tamminga, Astrid de Wind, Angelique E. de Rijk, Michiel A. Greidanus

**Affiliations:** 1 Department of Public and Occupational Health, Amsterdam UMC Location University of Amsterdam, Meibergdreef 9, Amsterdam, The Netherlands; 2 Societal Participation & Health, Amsterdam Public Health Research Institute, Amsterdam, The Netherlands; 3 Department of Social Medicine, Faculty of Health, Medicine and Life Sciences, Research Institute Primary Care and Public Health (CAPHRI), Maastricht University, Duboisdomein 30, Maastricht, The Netherlands

**Keywords:** Small business, Occupational health services, Sick-leave, Employer, Return to work, Online intervention, Intervention development

## Abstract

**Purpose:**

Employers of small and medium-sized enterprises (SMEs) face challenges in supporting employees on long-term sick-leave, due to limited resources and expertise available. This study aimed to develop an intervention assisting employers of SMEs in supporting long-term sick-listed employees during sick-leave and return to work (RTW).

**Methods:**

Intervention mapping (IM) steps 1–4 were employed to develop the intervention. For the needs assessment, 20 employers, 8 employees, 8 occupational physicians, and 9 other stakeholders were interviewed (step 1). A logic model of change was developed (step 2), followed by the identification of theoretical methods for achieving the changes required (step 3). The intervention was composed (step 4), incorporating the results of a pilot test with 4 employers, 4 employees, 4 occupational physicians, and 3 other stakeholders.

**Results:**

Identified needs (step 1) span knowledge on legislation, communication skills, stakeholder engagement, practical support, actions regarding RTW, relapse prevention, and organizational policy. Using the self-determination theory as the theoretical basis for improving employer intention and ability to support sick-listed employees (steps 2 and 3), a web-based intervention was developed (step 4) (hereafter: SME tool). The SME tool includes succinct tips, communication videos, and practical checklists. Minor adjustments were made following the pilot test, such as adding supplementary information on privacy regulations and preventive strategies.

**Conclusion:**

By focusing on enhancing SME employers' intention and ability to support their long-term sick-listed employee(s), the developed SME tool has the potential to improve the satisfaction of employees with the sick-leave and RTW support of their employer during long-term sick-leave.

## Introduction

Every year, millions of employees worldwide face work absenteeism due to health reasons, with the duration of sick-leave increasing in recent years [1–3]. Long-term sick-leave, generally defined as a period exceeding 4 to 8 weeks [3, 4], can lead to high costs for the employer, and society at large, due to, e.g., extended salary payments, costs of work accommodations, and hiring replacements [3–5]. Apart from providing financial security and structure, work provides social interactions and contributes to a person’s quality of life [6–8]. For many employees who are long-term or chronically ill, work remains an important part of their lives [9].

The key stakeholders in the RTW process are the sick-listed employee and the employer [10, 11]. Employees have the responsibility of adhering to medical advice, communicating with the employer, and when possible, actively engaging in RTW [10, 11]. Complementary, employers are in a position to accommodate the RTW and provide appropriate arrangements for employees [12–14]. As opposed to most countries, these responsibilities of employers are enforced by legislation in the Netherlands [11]. Nonetheless, providing the necessary support for employees on sick-leave proves challenging for employers [15–18]. In the past, there has been substantial attention to supporting employees during RTW, with many interventions available [19, 20], whereas employers received less attention, or only in relation to RTW of employees with a specific diagnosis such as cancer [21]. To bridge this gap, we aim to address the needs of employers in supporting employees on long-term sick-leave due to a wide range of health conditions.

Especially employers of small and medium-sized enterprises (SMEs) express a need for skills and knowledge to offer adequate support to employees on sick-leave, including communication skills and knowledge of applicable legislation [15]. SMEs often have limited human and financial resources to cover for sick-listed employees or make appropriate work adaptions [22]. They often do not have an HR department and lack organizational policies and experience with sick-leave guidance [15, 22, 23]. The approach toward sick-listed employees in SMEs is generally informal, relying on close relations between employees and employers [22, 24]. There is some evidence from Norway and Sweden that SMEs tend to use occupational health services (OHS) less frequently than larger organizations [25]. This may be because of financial limitations, which are often less restrictive for large enterprises [25]. However, when provided with support in case of sick-leave and RTW, SMEs generally consider OHS valuable [26]. The distinct challenges and characteristics of SMEs warrant attention in addressing sick-leave and RTW, as the implications can lead to unsustainable (financial) pressure on the organization and sick-listed employees [24]. Therefore, without attributing employer behavior to a lack of involvement, there is potential for improvement toward employee support. Consequently, we advocate for developing an intervention to support SME employers.

To enhance the likelihood of the intervention’s effectiveness, it is recommended to adopt a systematic approach and evidence base to intervention development [27]. This entails using existing knowledge, while gathering additional insights for the specific target group. Every intervention presupposes a behavioral change within a specific target group, necessitating a structured approach. Intervention mapping (IM) is preferable, because of its theoretical approach by focusing on behavioral change and its proven effectiveness in health promotion interventions [28, 29]. IM has also previously been used as a systematic approach for developing employer-based interventions [21, 30, 31]. In one of these previous studies, the web-based MiLES intervention was developed to support employers with the sickness absence guidance and RTW of employees with cancer [21]. A study on its use and perceived usefulness among employers suggested positive results [32]. The purpose of the present research was to develop a web-based intervention targeting SME employers, with the aim of supporting long-term sick-listed employees during sick-leave and RTW, using MiLES as a starting point and applying the IM protocol.

## Methods

### Design

**Fig. 1:**
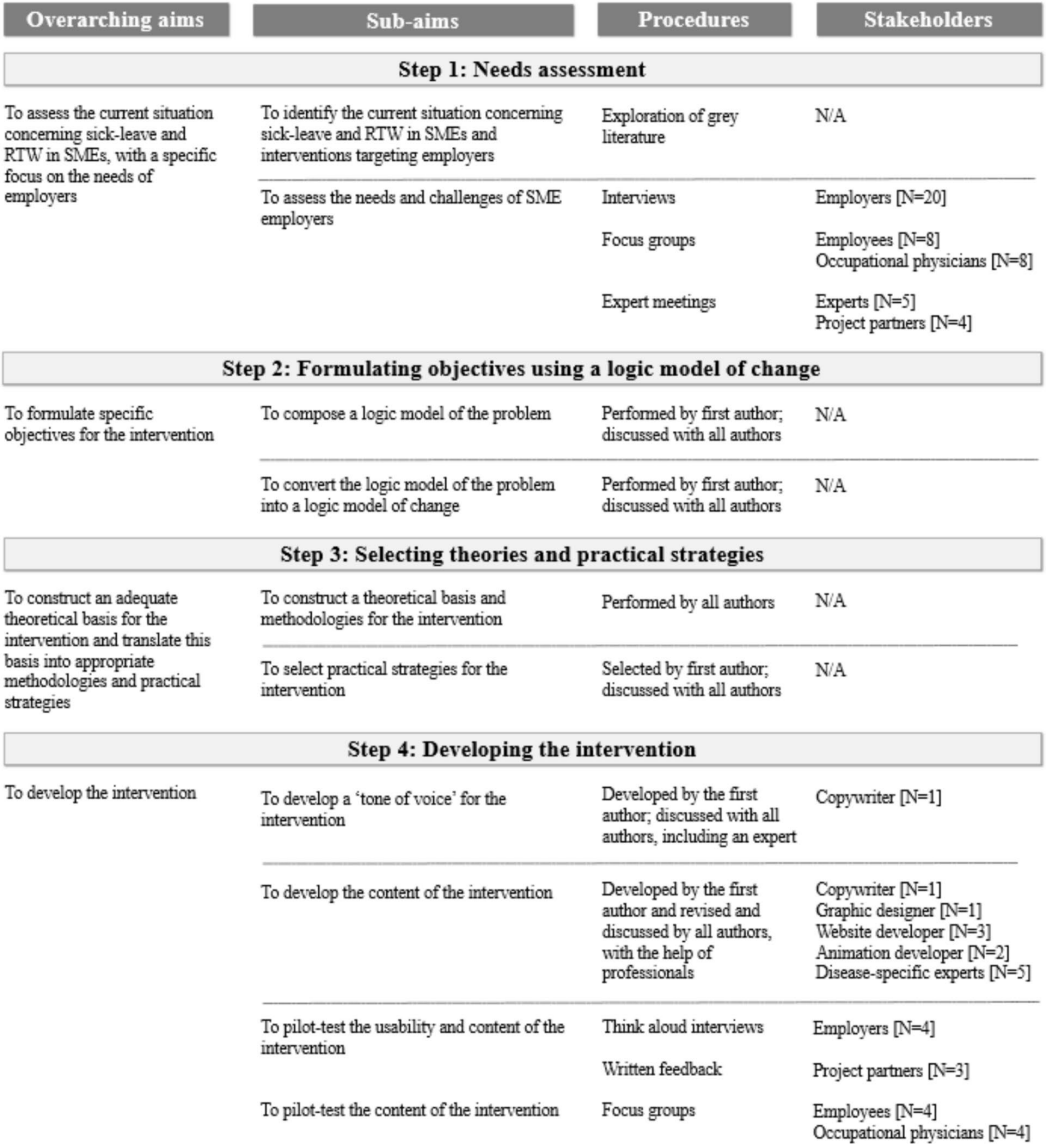
Overview of each IM step: overarching aim, sub-aims, procedures, and stakeholders involved in each step of the development of the intervention

The development of the intervention followed the IM protocol [29]. The IM protocol consists of six steps: (1) needs assessment; (2) formulating intervention objectives; (3) selecting theoretical methods and practical strategies; (4) developing the intervention; (5) planning for program adoption and implementation; and (6) planning for evaluation. This paper describes up and until the development of the intervention, i.e., IM steps 1 to 4, conducted from May 2022 to February 2024. IM steps 5 and 6 will be conducted in 2024 and 2025, according to the protocol that has been published [33], and will be published separately. Figure 1 provides an overview of the sequential steps, along with the involved stakeholders.

### Study Setting of the Netherlands

The development of the intervention took place in the Netherlands, where employers, by law, have a relatively large responsibility for sick-leave support. Specific characteristics are that RTW activities extent during the initial 2 years of sick-leave [10, 11]. The exact responsibilities of both employers and employees are outlined in the ‘Gatekeeper Improvement Act,’ underlining the importance of a structured RTW and the joint responsibility of employees and employers [17, 20]. For example, an occupational physician must be involved in long-term sick-leave of employees. Their role includes translating employees’ medical restrictions into work adjustments. In terms of work units, SMEs are defined as organizations with < 250 employees by the European Commission [34]. For the purpose of this study, SME employers are defined as individuals within the organization responsible for supporting employees during sick-leave and RTW, such as the owner of the enterprise or a direct supervisor.

### Pre-IM: Establishing the Foundation

The MiLES intervention, a web-based intervention assisting employers during the RTW of cancer survivors [21], has been employed as a foundation of the intervention development. Therefore, certain aspects, including mode of delivery (i.e., web-based), were predetermined and not subject to discussion during the development of this intervention. For all IM steps of this intervention, the applicability of the results of the specific IM step of the MiLES intervention was assessed through discussions with all authors.

### IM Step 1: Needs Assessment

The first sub-aim of the needs assessment was to identify the current situation concerning sick-leave and RTW in SMEs and interventions targeting employers. This was conducted via exploration of gray literature. The second sub-aim involved identifying the needs of SME employers regarding sick-leave support and RTW of employees on long-term sick-leave. This was conducted via individual interviews with SME employers, two focus groups with employees and two focus groups with occupational physicians. The primary needs were determined based on the recurring themes in both interviews and literature, and concerns that could be addressed effectively through a web-based intervention. To formulate the logic model of the problem, needs and problems were addressed through collaborative meetings among the authors and discussions with experts.

#### Interview and Focus Group Participants and Procedure

The participants for the interviews and focus groups were purposefully sampled through professional networks and project partners. The interviews and focus groups were conducted using a semi-structured topic list, with a focus on the problems and needs of SME employers with regard to sick-leave and RTW, and preferences of SME employers about the content of the intervention. Interviews and focus group sessions were conducted online via Teams and varied in duration from 1 to 2 h. Informed consent was obtained from all participants. All interviews and focus groups were audio-recorded and transcribed verbatim and analyzed using a thematic content analysis. The interviews were conducted by one researcher (DB), and the focus groups were moderated by two researchers (DB and MG).

### IM Step 2: Formulating Objectives Using a Logic Model of Change

In the second step of IM, a transition was made from the logic model of the problem to the logic model of change, assessing who and what should change to achieve the desired goal. Based on the results of IM step 1, the formulation of the objectives and the development of the logic model of change was conducted together with all authors.

### IM Step 3: Selecting Theories and Practical Strategies

In the third step of IM, appropriate theoretical methods and practical strategies, aligning with the change objectives formulated in step 2, were identified. The selection of suitable theories resulted from discussions involving all authors and literature on theoretical methods [29].

### IM Step 4: Intervention Production

In the fourth step of IM, the aim was to synthesize and use the outcomes from IM steps 1–3 into the development of the intervention. The content was initiated by the first author and revised by all authors. The technological features of the intervention were created by a professional web developer, in accordance with the research team. To ensure the intervention’s quality, we furthermore enlisted the expertise of a professional copywriter, an animation developer, and disease-specific experts. Throughout the development, collaborative brainstorming sessions were conducted with the research team to refine the content and layout of the intervention.

Finally, a pilot test of the intervention’s usability and content was conducted. Figure 1 provides an overview of all involved participants and stakeholders. Employers tested the intervention, through think-aloud interviews in which participants gave their opinion on its usability and content. Employees and occupational physicians gave their opinion on the content during focus groups. Additionally, several project partners used the intervention and provided written feedback on its usability and content. Interviews were audio-recorded, and reviewed, with suggestions noted and categorized by topic. A suggestion was implemented if it met one or more of the following criteria: (1) aligns with the scope and purpose of the intervention, (2) complies with Dutch legislation, and (3) is feasible within the project duration and budget.

The study protocols for IM step 1 (METC2022.0160) and 4 (METC2023.0727) have been reviewed by the Medical Ethical Committee of the Amsterdam University Medical Center (Amsterdam UMC), the Netherlands. The METC has exempted the study protocols from ethical review, according to the Dutch Medical Research Involving Human Subjects Act (WMO).

## Results

### Step 1: Needs Assessment

From the exploration of gray literature and the interviews and focus groups, it appeared that in general, within SMEs, there are fewer resources and expertise (e.g., knowledge, financial means, skills) available to provide support to employees compared to large enterprises. The owner of the SME, often overseeing all HR responsibilities, may not be an expert in all domains. For that reason, the intervention should primarily focus on addressing information needs regarding legislation, enhancing employer autonomy, strengthening communication skills, providing tailored support for various health conditions, and alleviating the employer’s responsibilities.

**Fig. 2:**
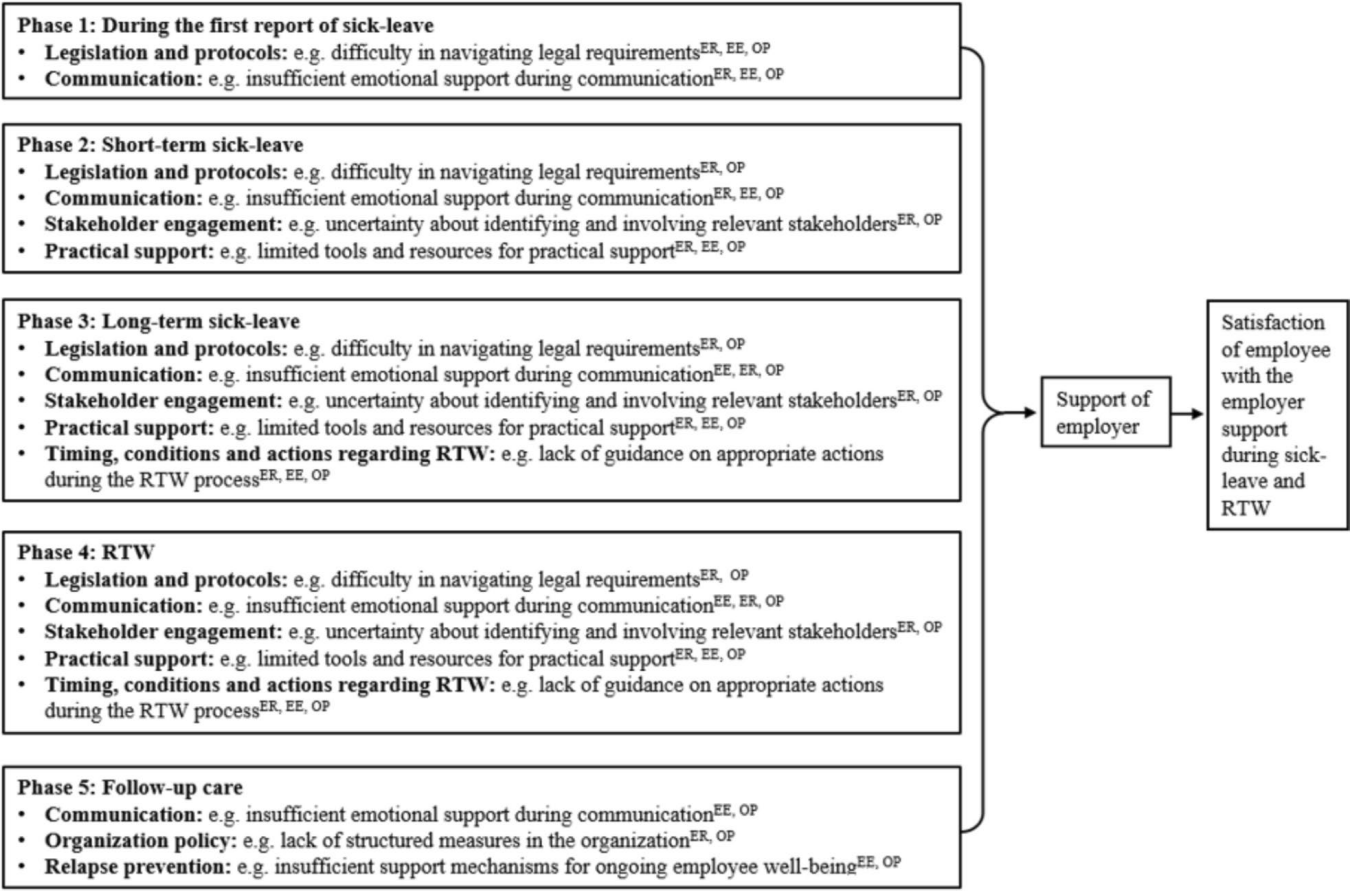
Logic model of the problem. *ER* Expressed employers, *EE* Expressed employees, *OP* Expressed occupational physicians

Based on the interviews and focus groups, five different phases of the RTW process were identified: (1) around first report of sick-leave; (2) short-term sick-leave; (3) long-term sick-leave; (4) RTW; and (5) follow-up care. Moreover, the importance for disease-transcending information was emphasized, including a focus on specific needs of the most prevalent diagnoses registered in case of long-term sick-leave. Additionally, topics such as understanding legislation and its implications, as well as engaging with key stakeholders, were identified as typical needs for SME employers. Figure 2 summarizes the main results of the challenges faced by SME employers in supporting employees on long-term sick-leave, through a logic model of the problem with seven problem domains: (1) legislation; (2) communication (including emotional support); (3) stakeholder engagement; (4) practical support (including job accommodation); (5) timing, conditions and actions regarding RTW; (6) relapse prevention; and (7) organization policy. Recognizing diverse priorities in each phase, the majority of needs were identified by all interviewees. Among employees, emotional support was perceived as more important, while aspects such as legislation and stakeholder engagement were perceived as less critical. Employers focused on the employee’s RTW during sick-leave and prioritized subsequent follow-up care less. Certain needs, such as the need for peer support, the availability of someone to discuss acute issues with, and the need for financial support, were not included in the logic model of the problem, as they were considered beyond the functionalities of a public (non-password protected) web-based intervention.

The logic model of the problem (Fig. 2) underscores the need for increased assistance of employers, with the ultimate aim to improve satisfaction of the sick-listed employee with the employer support. As highlighted by employers themselves, many external factors beyond their support influence the actual RTW of their employees (e.g., severity of the disease). Therefore, satisfaction of employees with the employer support during sick-leave and RTW emerged as a more appropriate primary aim of the intervention compared to actual RTW.

### Step 2: Formulating Objectives Using a Logic Model of Change

**Fig. 3:**
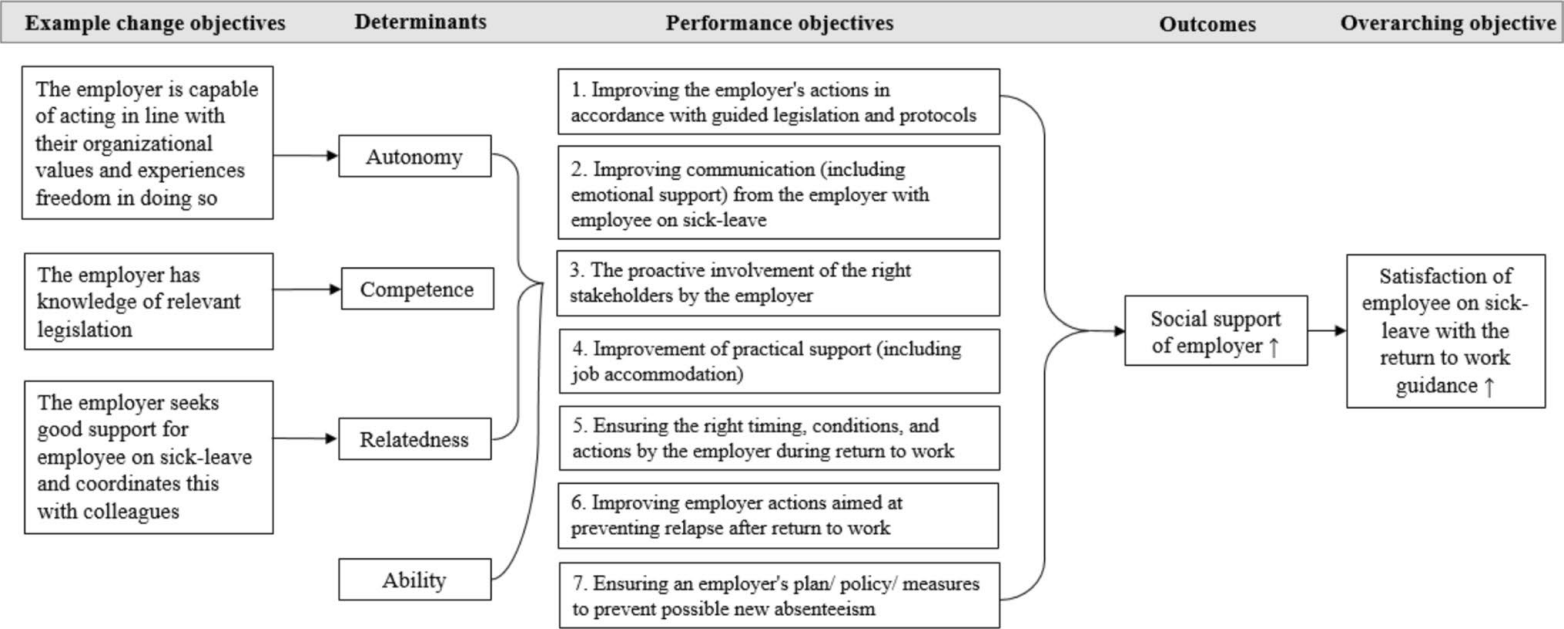
Logic model of change

The logic model of change shows the pathways for program effects (Fig. 3). The matrices describe the most immediate change to be addressed by the intervention (“Appendix 1”). To stimulate the ideal employer behavior, the self-determination theory (SDT), a theory of human motivation, is used as a theoretical strategy to increase employer intention to support employees on long-term sick-leave [35]. It states that all individuals share three basic psychological needs. If these needs are addressed, the person’s motivation or intention to achieve goals is affected positively. In the application of this theoretical strategy for this study, the first component, *autonomy*, involves acting in line with personal and organizational values, fostering a sense of freedom in choices. It does not imply independence from others, but rather an inherent inclination of an individual to experience individual freedom and a sense of self-determination. Applying this component to our aim, we might develop an intervention that improves the employer’s intention to support his or her employee’s RTW by acknowledging the autonomy of the employer. We assumed that acknowledging autonomy would fit with the general attitude of employers of SMEs. For example, the employer is capable of acting in line with their values within the legal obligations and experiences freedom in doing so. The second component, *relatedness*, emphasizes the relationship dynamics in the workplace. When this component is addressed by the intervention, the positive aspects of the relationship of the employer, employee, and other colleagues are emphasized. This is assumed to have a positive effect on the employer to address RTW support and directly impact the employee in the perceived support. For example, the employer seeks good support for employees and coordinates this with colleagues. The third component, *competence*, includes referring to knowledge and skills as encouragement for supporting RTW. We also assumed that in order to actually give RTW support, the employer might need additional information. For example, the employer has knowledge of relevant legislation to ensure competence in the field. Lastly, *ability* was added as a determinant alongside *autonomy*, *competence*, and *relatedness*. *Ability* refers to the resources and possibilities available for the employer to provide support, such as the capacity to make necessary work adjustments.

### Step 3: Selecting Theories and Practical Strategies

#### Theoretical Basis

Using the SDT as the leading theory within the intervention, the researchers chose eleven additional suitable theoretical methods [29]. Table 1 presents an overview of all chosen theoretical methods for the intended progress concerning the employer’s behavior change. Firstly, to enhance *autonomy*, methods such as enhancing network linkages and modeling were selected (e.g., [36, 37]). Enhancing network linkages helps employers connect with reliable resources and professionals, while modeling offers examples of good employer practices, providing autonomy-supportive guidance. Secondly, given that many SME employers lack formal HR training, methods such as active learning, advanced organizers, elaboration, and tailoring were chosen to enhance *competence* (e.g., [38–40]). For instance, active learning and elaboration aim to improve knowledge on supporting sick-listed employees, while tailoring may help to make the provided tips and resources relevant to the current sick-leave phase or reason of sick-leave of the employee. Thirdly, to foster a supportive relationship during sick-leave, methods such as empathy training and mobilizing social support were chosen to enhance *relatedness* (e.g., [36, 41]). These methods are thus in line with intrinsically motivating SME employers to support the RTW of their employee.

**Table 1:** Overview of theoretical methods and practical strategies

SDT component	Theoretical methods	Practical strategies
Autonomy	• Enhancing network linkages • Modeling	• Use of resilience-enhancing language, design and images • Examples of good employer practices
Competence	• Active learning • Advance organizers • Elaboration • Facilitation • Feedback • Tailoring	• Succinct, tailored tips and information • Animations and videos • Provision of checklists and fill-in templates • Links to reliable external sources
Relatedness	• Empathy training • Mobilizing social support • Shifting perspective	• Encouragement of proactive communication • Quotes of employers • Animations and videos

#### Practical Strategies

Table 1 presents an overview of all aligning practical strategies for the intended progress concerning the employer’s behavior change. For example, the advanced organizer method helps the employer to comprehend the RTW steps and to make an action plan through the practical strategies of a fill-in template.

### Step 4: Intervention Production

#### The Intervention

**Fig. 4:**
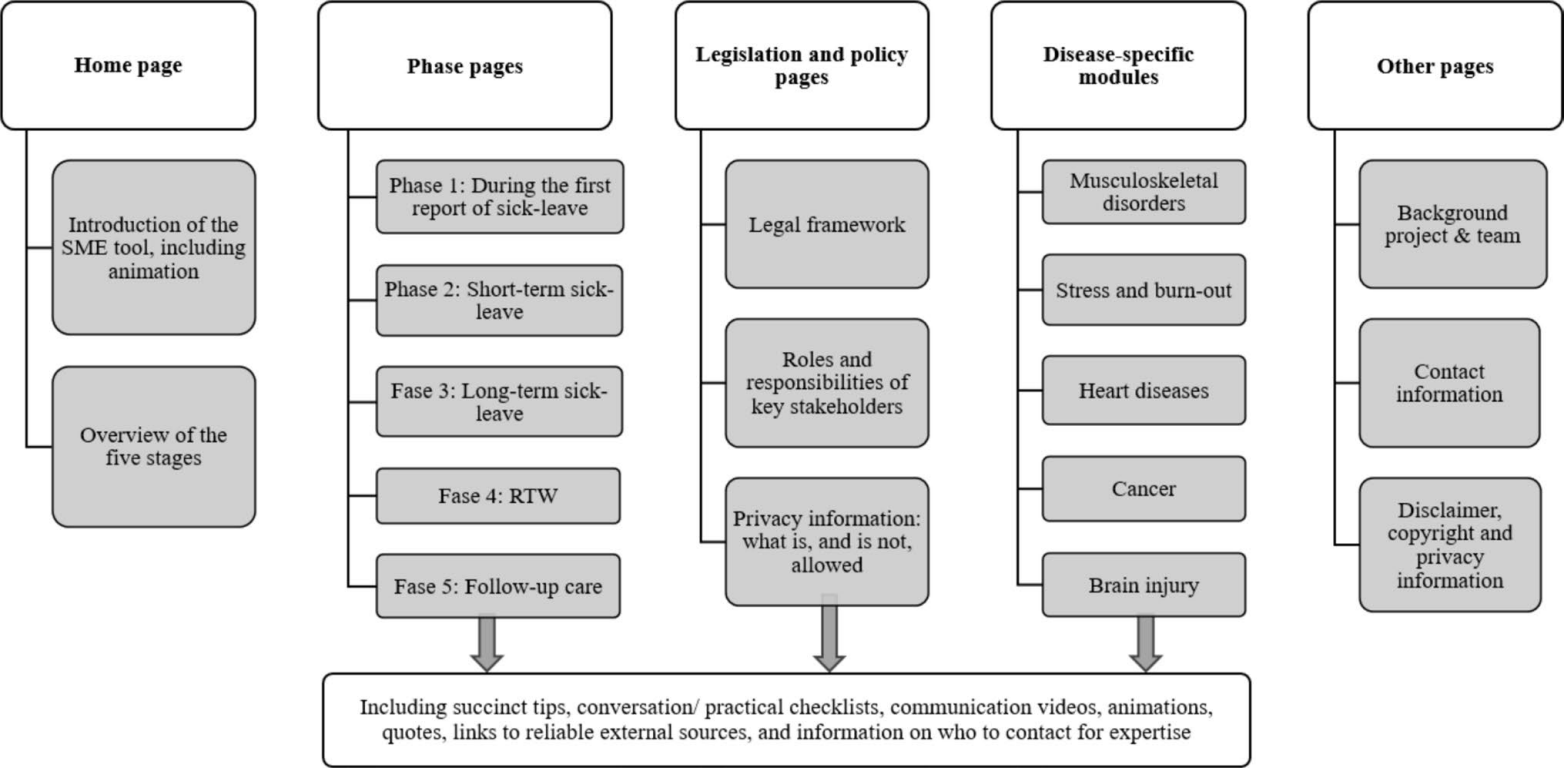
Page structure and content of the SME tool

The results of the previous IM steps were operationalized in the web-based intervention, that is, the SME tool. The main outline of the page structure and content is depicted in Fig. 4. The tool consists of 19 different webpages, each averaging approximately 1000 words. Employers can select the specific phase of the RTW process of their current employee for tailored information. The pages within the SME tool include succinct information and practical tips, communication videos, conversations checklists, quotes from SME employers, and links to reliable external sources. To enhance accessibility, each page is accompanied by print buttons, allowing employers to generate hard copies for their records or offline reference. The textual content within the SME tool is written in practical and understandable language. A detailed breakdown of specific webpages is provided in “Appendix 2.”

#### Pilot Testing the SME Tool

The SME tool was perceived as a clear, user-friendly, and complete tool in the pilot test. All participants recognized it as a valuable tool for employers. Following this pilot test, the researchers made minor adjustments to the tool, such as textual changes, enhancing the video experience, and adding supplementary information on some pages. This supplementary information included details on privacy regulations, where to find preventive strategies, and elaboration of specific RTW situations, among other relevant topics. With these adjustments, the final version of the SME tool 1.0 was developed. Some suggestions from participations did not meet the criteria for incorporation in the SME tool, i.e., adding an extra page featuring trends in average absenteeism rates, guidelines for recovery, and support for workplace conflicts between employer and employee. Although outside of the scope of sick-leave support, some referrals to external resources and professionals that provide tailored, one-on-one support for workplace conflicts and financial subsidies were included in the SME tool.

## Discussion

This paper describes the development process of the SME tool using the first four steps of IM. The tool is a web-based intervention aimed at improving the satisfaction of long-term sick-listed employees with the support from their employer, by improving the intention and ability of SME employers to provide appropriate support during sick-leave and RTW. The SME tool consists of succinct information and practical tips, communication videos, conversations checklists, quotes, and links to reliable external sources.

The seven problem domains deriving from the needs assessment, i.e., knowledge on legislation, communication skills, stakeholder engagement, practical support, actions regarding RTW, relapse prevention, and implementation of organizational policy, align with existing international literature on the challenges of SME employers [15, 22, 23]. For example, even though SME employers mostly lack HR training, they are often responsible for the implementation of many HR practices [42, 43]. Our needs assessment corroborated this, showing that SME employers have many responsibilities and seek support in HR duties. When examining existing web-based interventions, few tools were identified for SMEs in occupational health context. In the United Kingdom, the SART tool was developed, a tool designed to support SMEs in monitoring sickness absence duration and providing advice and best practices for supporting employees to RTW [44]. Although the objective may differ, recommendations from the SART participants, such as advocating for a web-based format and links to other support systems, align with the features of the SME tool. However, some recommendations of the SART tool, such as establishing local support networks and offering of free or low-cost training tailored to SMEs, were not feasible within the scope of the SME tool and the project, and thus not incorporated. Additionally, the PROWORK intervention, also developed using the IM approach, shares similarities with the SME tool [31]. PROWORK includes a toolkit for employers that focuses on supporting employees on long-term sick-leave, particularly those with mental health challenges [31]. Both the SME tool and PROWORK are theoretically grounded and emphasize communication between employees and employers. However, PROWORK includes three 1-h coaching sessions to facilitate engagement, whereas the SME tool relies on a self-guided, web-based format with videos, checklists, and tips—an approach that may better suit the needs and nature of SMEs. This may indicate that the IM approach allows for developing interventions tailored to the specific characteristics and needs of the target group.

While the foundation of the SME tool was based on the MiLES tool, a tool targeting employers during the RTW of cancer survivors [21], the SME tool distinguishes itself as a disease-transcending tool specifically targeted at SME employers. Certain aspects were the same, such as the framework of the web-based intervention, but many outcomes of the IM steps were different. For example in IM step 1, the needs assessment, five different phases of the RTW process, were identified, as opposed to the four phases of the MiLES tool. Furthermore, the MiLES tool used the trans-theoretical model of change, while we have chosen for the SDT theory as this suits the SME context best in our opinion [45]. The suitability for SMEs is an aspect we consider important, given the frequent misalignment of interventions in SMEs with regard to health and safety [46]. Additionally, as opposed to the MiLES tool, the distinct characteristics of the SME environment prompted the need for information on legislation and involved stakeholders, which were developed and included in the SME tool in step 4.

The use of the MiLES tool [21] as a starting point is the first strength of our research, namely the use of an existing, positively received intervention among employers. Secondly, through the structured development following the IM steps, we tailored our tool to be disease-transcending and align with the needs of SME employers. By using gray literature and active engagement with stakeholders, the development aimed to comprehensively address the challenges associated with sick-leave in SMEs. This is exemplified in the needs assessment, which showed that insights from different perspectives per identified phase differed between employers and employees. Another strength of our study is the pilot-testing phase. We were able to gather valuable feedback and refine the tool accordingly, by involving employers, employees, occupational physicians, and project partners in testing the prototype. Finally, the SME tool marks the first systematically developed tool that is aimed at SME employers in the Netherlands. The tool therefore addresses an important research gap, as identified in the needs assessment that SME employers lack the resources and expertise to support sick-listed employees. Although developed in and for the Dutch context, the SME tool has potential for adaptation and translation to other countries. Beyond variation at legislation level (e.g., the specific role of the occupational physician), many fundamental aspects of the tool hold relevance internationally. For example, while legal obligations for employers regarding employees on sick-leave may differ, effective communication is considered good practice by employers across eight European countries [47], thus making the essence of the tool translatable to other countries.

Some limitations should also be considered. Firstly, while the SME tool tackles several challenges faced by employers, it does not address certain issues that are outside the scope of sick-leave support. These include the factors as financial aspects and conflicts in the workplace, which may be too complex for a website to address effectively. The SME tool addresses this need by referring to these resources and professionals that provide tailored, one-on-one support for workplace conflicts and financial subsidies. Functionalities such as a chat function for individual coaching or peer support mechanisms are also considered outside the scope of the SME tool. Secondly, the characteristics of a web-based intervention naturally impose limitations. While web-based interventions excel in providing information and examples, they lack the capacity to provide personalized feedback for evaluating skills [48].

Further research on the effectiveness of the SME tool is needed (IM steps 5 and 6). As external factors, such as sufficient time and resources, may also contribute to the success rate of the SME tool, a comprehensive process evaluation is also recommended to interpret the results on effectiveness.

## Conclusion

Recognizing the diversity of health reasons for long-term sick-leave, there is a need for a supportive disease-transcending intervention, tailored to the specific challenges encountered by employers of SMEs. This study contributes to this need, by describing the systematic development of the SME tool, a web-based intervention aiming to improve the intention and ability of SME employers to provide appropriate support during sick-leave and RTW for their long-term sick-listed employees.

## Figures and Tables

**Fig. 5:**
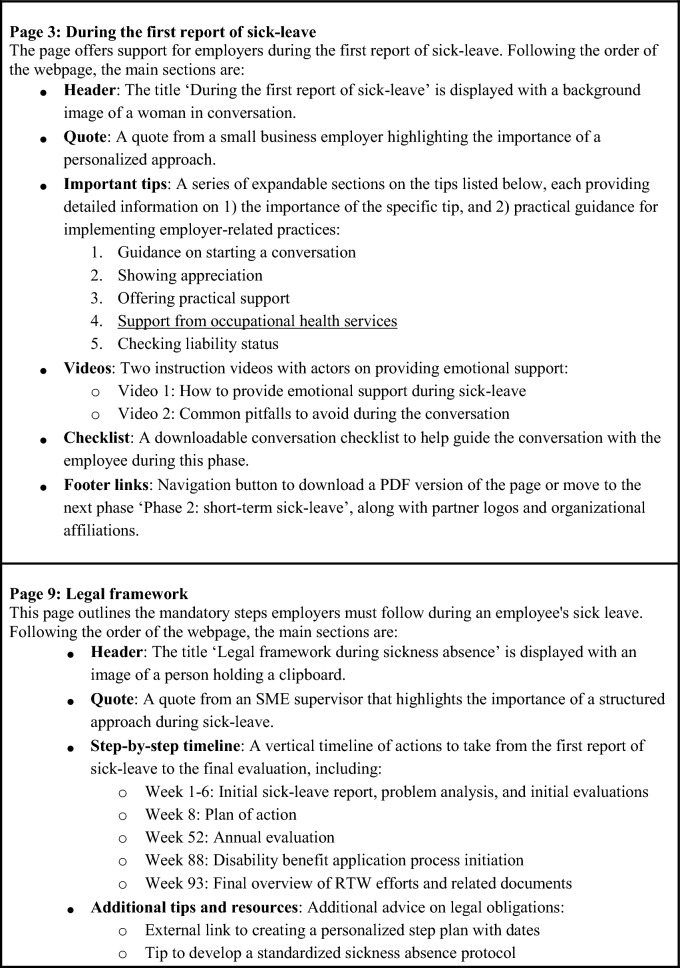

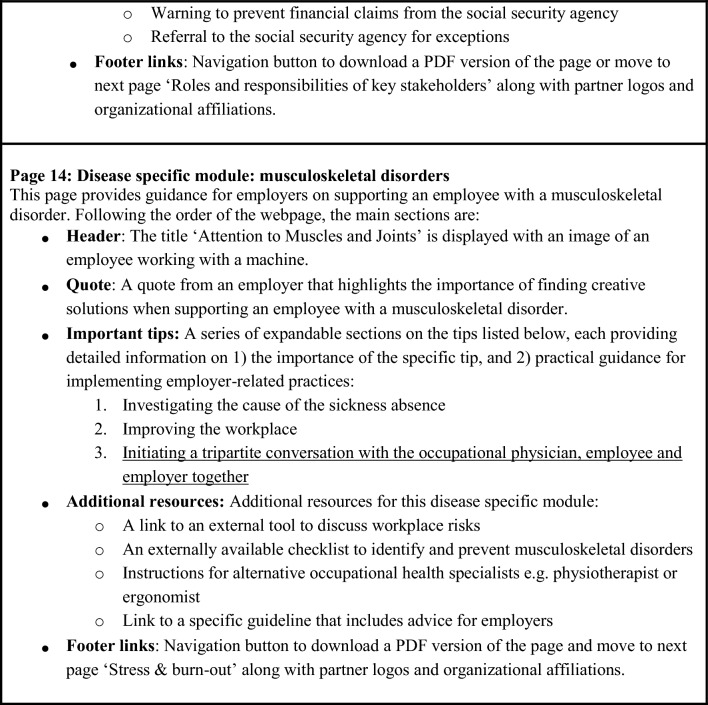
Breakdown of pages 3, 9, and 14 of the SME tool

## Data Availability

Data sharing is possible upon reasonable request. Not applicable.

## Appendix 1

See Tables 2, 3, 4, 5, and 6.

**Table 2:** Matrix of change objectives regarding phase 1: around first report of sick-leave

Performance objectives	Determinants		
	Autonomy	Competence	Relatedness
1.1 Improving the employer's actions in accordance with guided legislation and protocols	1.1.1 The employer is able to apply knowledge of legislation in accordance with the company’s values 1.1.2 The employer experiences flexibility to act in the spirit of the law 1.1.3 The employer feels resilient to make the initial decisions following sick-leave in accordance with the company’s values 1.1.4 The employer is able to create sick-leave records in accordance with the company’s values	1.1.5 The employer has knowledge of relevant legislation 1.1.6 The employer has skills to act according to protocols 1.1.7 The employer has an overview of the steps in the sick-leave and RTW process 1.1.8 The employer has the skills to create sick-leave records 1.1.9 The employer knows when and how to access the Employee Insurance Agency (in Dutch: UWV) website	1.1.10 The employer ensures that initial decisions and mutual expectations are coordinated with the employee and in accordance with legislation 1.1.11 The employer relieves the employee of administration 1.1.12 The employer makes it clear that the employer and employee share responsibility
1.2 Improving communication (including emotional support) from the employer with employees on sick-leave	1.2.1 The employer feels resilient and experiences freedom in communicating with the employee 1.2.2 The employer is able to share information with colleagues in accordance with the company’s values 1.2.3 The employer experiences flexibility in establishing a strong foundation for sick-leave counseling	1.2.4 The employer is able to communicate with the employee following sick-leave (e.g., responding constructively to different diagnoses) 1.2.5 The employee knows what he/she may or may not ask 1.2.6 The employee is able to coordinate with the employee who is allowed to know what 1.2.7 The employee can gather sufficient information from the employee for initial decisions following sick-leave	1.2.8 The employer is sympathetic and open to the employee to create a strong foundation for sick-leave support 1.2.9 The employer gives personal attention to the employee and recognition for the illness 1.2.10 The employer can cope with the different roles toward the employee (e.g., as employer or friend) 1.2.11 The employer understands when the employee needs more interaction or space 1.2.12 The employer makes the employee feel he/she is not alone during the sick-leave and RTW process

**Table 3:** Matrix of change objectives regarding phase 2: short-term sick-leave

Performance objectives	Determinants		
	Autonomy	Competence	Relatedness
2.1. Improving the employer's actions in accordance with guided legislation and protocols	2.1.1 The employer is able to apply knowledge of legislation in accordance with the company’s values 2.1.2 The employer experiences flexibility to act in the spirit of the law 2.1.3 The employer feels resilient to meet financial obligations 2.1.4 The employer is able to create sick-leave records in accordance with the company’s values	2.1.5 The employer has knowledge of relevant legislation 2.1.6 The employer has skills to act according to protocols 2.1.7 The employer has an overview of the steps in the sick-leave and RTW process 2.1.8 The employer has the skills to create sick-leave records 2.1.9 The employer knows when and how to access the Employee Insurance Agency (in Dutch: UWV) website	2.1.10 The employer ensures that initial decisions and mutual expectations are coordinated with the employee and in accordance with legislation 2.1.11 The employer relieves the employee of administration 2.1.12 The employer makes it clear that the employer and employee share responsibility
2.2 Improving communication (including emotional support) from the employer with employees on sick-leave	2.2.1 The employer feels resilient and experiences freedom in communicating with the employee 2.2.2 The employer is able to share information with colleagues in accordance with the company’s values 2.2.3 The employer is able to communicate effectively in accordance with their own and company’s values	2.2.4 The employer is able to communicate with the employee during the first six weeks of sick-leave (e.g., knows what or what not to ask and when and how to talk about work with the employee) 2.2.5 The employer is able to coordinate with the employee who is allowed to know what 2.2.6 The employer is able to make agreements with the employee	2.2.7 The employer contributes to creating a safe and open work atmosphere 2.2.8 The employer gives personal attention to the employee and recognition for the illness 2.2.9 The employer can cope with the different roles toward the employee (e.g., as employer or friend) 2.2.10 The employer understands when the employee needs more interaction or space 2.2.11 The employer takes initiative in contacting the employee 2.2.12 The employer makes the employee feel he/she is not alone during the sick-leave and RTW process
2.3. The proactive involvement of the right stakeholders by the employer	2.3.1 The employer experiences flexibility in making decisions regarding engaging stakeholders 2.3.2 The employer does not feel overwhelmed by all the different stakeholders 2.3.3 The employer feels resilient in engaging and maintaining contact with other stakeholders	2.3.4 The employer has knowledge of what stakeholders, such as an occupational physician, have to offer 2.3.5 The employer has insight into other potentially effective interventions and support 2.3.6 The employer is able to effectively approach the right stakeholders at the right time 2.3.7 The employer understands the importance of proactively engaging the right stakeholders 2.3.8 The employer is able to formulate questions for, e.g., the occupational physician	2.3.9 The employer communicates and collaborates closely with stakeholders, such as occupational physician 2.3.10 The employer understands the importance of having good relationships with various stakeholders 2.3.11 Depending on the needs, the employer discusses the involvement of stakeholders with the employee
2.4. Improvement of practical support	2.4.1 The employer feels resilient and remains autonomous in facilitating practical support 2.4.2 The employer can cope with the unpredictability and does not feel vulnerable as a company 2.4.3 The employer feels resilient in arranging substitution for the employee or is able to accept that work will remain unfinished 2.4.4 The employer is able to find solutions aligning with the company for practical obstacles 2.4.5 The employer feels resilient to ask for and receive support from colleagues	2.4.6 The employer has knowledge of possibilities for practical support 2.4.7 The employer understands the importance of practical support 2.4.8 The employer recognizes practical obstacles for the prognosis of the employee 2.4.9 The employer has knowledge on the cost-effectiveness of practical support 2.4.10 The employer increases the implementation of practical support	2.4.11 In consultation with the employee, the employer relieves the employee in work tasks and arranging replacement 2.4.12 The employer seeks appropriate practical support for the employee 2.4.12 The employer communicates with colleagues about the consequences and solutions of the sick-leave for the company 2.4.13 The employer maintains good contact with other colleagues about practical support

**Table 4:** Matrix of change objectives regarding phase 3: long-term sick-leave (only additional to 2)

Performance objectives	Determinants		
	Autonomy	Competence	Relatedness
3.1 Improving the employer's actions in accordance with guided legislation and protocols	See phase 2	3.1.10 The employer has the skills to regularly evaluate the agreements and progress of the employee	See phase 2
3.2. Improving communication (including emotional support) from the employer with employees on sick-leave	See phase 2	3.2.8 The employer has the knowledge and skills on long-term, complex sick-leave and RTW support	3.2.14 The employer deals well with employees who withhold contact 3.2.15 The employer continues to show understanding as the sick-leave continues
3.3. The proactive involvement of the right stakeholders by the employer	3.3.3 The employer is able to retain autonomy if the sick-leave guidance is taken over by the occupational health service	See phase 2	See phase 2
3.4 Improvement of practical support	See phase 2	See phase 2	See phase 2
3.5 Ensuring the right timing, conditions, and actions by the employer during return to work	3.5.1 The employer feels resilient and experiences freedom in looking for tailored solutions aligning with the company 3.5.2 The employer has a network of fellow companies to contact	3.5.3 The employer has knowledge of a normative framework regarding optimal RTW and can apply this (i.e., not RTW as soon as possible) 3.5.4 The employer has knowledge on how to organize RTW for the first and second track 3.5.5 The employer can meet RTW obligations, such as finding suitable work and maintaining RTW records 3.5.6 The employer understands the importance of sustainable RTW and work modifications	3.5.7 The employer coordinates RTW plans with the employee and other stakeholders 3.5.8 The employer protects the employee from returning to work too early 3.5.9 The employer lowers the threshold for RTW 3.5.10 The employer maintains good contact and coordinates work adjustments with other colleagues during RTW

**Table 5:** Matrix of change objectives regarding phase 4: RTW

Performance objectives	Determinants		
	Autonomy	Competence	Relatedness
4.1 Improving the employer's actions in accordance with guided legislation and protocols	4.1.1 The employer is able to apply knowledge of legislation in accordance with the company’s values 4.1.2 The employer experiences flexibility to act in the spirit of the law 4.1.3 The employer feels resilient to meet financial obligations 4.1.4 The employer is able to create sick-leave records in accordance with the company’s values	4.1.5 The employer has knowledge of relevant legislation 4.1.6 The employer has skills to act according to protocols 4.1.7 The employer has an overview of the steps in the sick-leave and RTW process 4.1.8 The employer has organized records of RTW efforts 4.1.9 The employer knows when and how to access the Employee Insurance Agency (in Dutch: UWV) website 4.1.10 The employer has the skills to regularly evaluate the agreements and progress of the employee	4.1.11 The employer ensures that initial decisions and mutual expectations are coordinated with the employee and in accordance with legislation 4.1.12 The employer relieves the employee of administration 4.1.13 The employer makes it clear that the employer and employee share responsibility
4.2 Improving communication (including emotional support) from the employer with employees on sick-leave	4.2.1 The employer feels resilient and experiences freedom in communicating with the employee 4.2.2 The employer is able to share information with colleagues in accordance with the company’s values 4.2.3 The employer is able to communicate effectively in accordance with their own and company’s values	4.2.4 The employer is able to communicate with the employee during the return to work 4.2.6 The employer is able to coordinate with the employee who is allowed to know what 4.2.7 The employer is able to make agreements with the employee 4.2.8. The employer has the knowledge and skills on long-term, complex sick-leave and RTW support 4.2.9 The employer is able to encourage the employee's enthusiasm for RTW 4.2.10 The employer is able to provide effective feedback 4.2.11 The employer is able to effectively discuss job satisfaction	4.2.12 The employer contributes to creating a safe and open work atmosphere 4.2.13 The employer gives personal attention to the employee and recognition for the illness 4.2.14 The employer can cope with the different roles toward the employee (e.g., as employer or friend) 4.2.15 The employer takes initiative in contacting the employee 4.2.16 The employer exerts no pressure to RTW 4.2.17 The employer maintains equal control as employee in RTW 4.2.18 The employer makes the employee feel he/she is not alone during the sick-leave and RTW process
4.3 The proactive involvement of the right stakeholders by the employer	4.3.1 The employer experiences flexibility in making decisions regarding engaging stakeholders 4.3.2 The employer does not feel overwhelmed by all the different stakeholders 4.3.3 The employer feels resilient in engaging and maintaining contact with other stakeholders 4.3.4 The employer is able to retain autonomy if the sick-leave guidance is taken over by the occupational health service	4.3.5 The employer has knowledge of what stakeholders, such as an occupational physician, have to offer 4.3.6 The employer has insight into other potentially effective interventions and support (e.g., a RTW coach) 4.3.7 The employer is able to effectively approach the right stakeholders at the right time 4.3.8 The employer understands the importance of proactively engaging the right stakeholders 4.3.9 The employer is able to formulate questions for, e.g., the occupational physician 4.3.10 The employer is able to understand and act on the advice of the occupational physician	4.3.11 The employer communicates and collaborates closely with stakeholders, such as occupational physician 4.3.12 The employer understands the importance of having good relationships with various stakeholders 4.3.13 Depending on the needs, the employer discusses the involvement of stakeholders with the employee
4.4 Improvement of practical support	4.4.1 The employer feels resilient and remains autonomous in facilitating practical support 4.4.2 The employer can cope with the unpredictability and does not feel vulnerable as a company 4.4.3 The employer feels resilient in arranging substitution for the employee or is able to accept that work will remain unfinished 4.4.4 The employer is able to find solutions aligning with the company for practical obstacles 4.4.5 The employer feels resilient to ask for and receive support from colleagues	4.4.6 The employer has knowledge of possibilities for practical support 4.4.7 The employer understands the importance of practical support 4.4.8 The employer recognizes practical obstacles for the prognosis of the employee 4.4.9 The employer has knowledge on the cost-effectiveness of practical support 4.4.10 The employer increases the implementation of practical support	4.4.11 In consultation with the employee, the employer relieves the employee in work tasks and arranging replacement 4.4.12 The employer seeks appropriate practical support for the employee 4.4.13 The employer communicates with colleagues about the consequences and solutions of the sick-leave for the company 4.4.14 The employer maintains good contact with other colleagues about practical support
4.5 Ensuring the right timing, conditions, and actions by the employer during return to work	4.5.1 The employer feels resilient and experiences freedom in looking for tailored solutions aligning with the company 4.5.2 The employer has a network of fellow companies to contact	4.5.3 The employer has knowledge of a normative framework regarding optimal RTW and can apply this (i.e., not RTW as soon as possible) 4.5.4 The employer has knowledge on how to organize RTW for the first and second track 4.5.5 The employer can meet RTW obligations, such as finding suitable work and maintaining RTW records 4.5.6 The employer understands the importance of sustainable RTW and work modifications	4.5.7 The employer coordinates RTW plans with the employee and other stakeholders 4.5.8 The employer protects the employee from returning to work too early 4.5.9 The employer lowers the threshold for RTW 4.5.10 The employer maintains good contact and coordinates work adjustments with other colleagues during RTW

**Table 6:** Matrix of change objectives regarding phase 5: follow-up care

Performance objectives	Determinants		
	Autonomy	Competence	Relatedness
5.1 Improving employer actions aimed at preventing relapse after return to work	5.1.1 The employer increases his/her resilience by personal development and self-reflection 5.1.2 The employer is able to effectively monitor the employee in accordance with company’s values	5.1.3 The employer understands the importance of follow-up care 5.1.4 The employer can effectively monitor the employee 5.1.5 The employer has insight into potential interventions and support for follow-up care 5.1.6 The employer understands the importance of reviewing the workplace together	5.1.7 The employer remains giving personal attention to the employee and his/her needs after RTW 5.1.8 The employer develops a long-term plan in consultation with the employee 5.1.9 The employer is in contact with other colleagues regarding the progress of the employee
5.2 Improving communication (including emotional support) from the employer with employees on sick-leave	5.2.1 The employer feels resilient and experiences freedom in communicating with the employee 5.2.2 The employer is able to communicate effectively in accordance with their own and company’s values	5.2.3 The employer is able to communicate with the employee during the follow-up care 5.2.4 The employer is able to make agreements with the employee 5.2.5 The employer is able to provide effective feedback 5.2.6 The employer is able to effectively discuss job satisfaction	5.2.7 The employer gives personal attention to the employee and recognition for the illness 5.2.8 The employer can cope with the different roles toward the employee (e.g., as employer or friend) 5.2.9 The employer takes initiative in contacting the employee 5.2.10 The employer creates extended follow-up mental support
5.3 Ensuring an employer's plan/policy/measures to prevent possible new absenteeism	5.3.1 The employer feels resilient to make plans to prevent new absenteeism in accordance with company’s values 5.3.2 The employer feels resilient in dealing with high absenteeism in the company 5.3.3 The employer is able to arrange sick-leave insurance in accordance with the company’s values 5.3.4 The employer has access to a network of fellow companies to contact if needed	5.3.5 The employer has an overview of the steps in the sick-leave and RTW process 5.3.6 The employer is able to establish a sick-leave protocol in alignment with the company 5.3.7 The employer has knowledge of and can apply preventative measure 5.3.8 The employer is able to reflect on his/her sick-leave and RTW support to the employee 5.3.9 The employer knows how to communicate and take action regarding the passing of an employee within the company	5.3.10 The employer contributes to creating a safe and open work atmosphere 5.3.11 The employer inquires among other colleagues about their needs to prevent new absenteeism 5.3.12 The employer produces an sick-leave protocol in consultation with the employees 5.3.13 The employer requests feedback from the employee on his/her support during sick-leave and RTW

## Appendix 2

See Fig. 5.
